# Supplementary material for: A generative learning model for saccade adaptation
Source: PLoS Comput Biol. 2019 Aug 9;15(8):e1006695. doi: 10.1371/journal.pcbi.1006695 (PMC6703699; doi:10.1371/journal.pcbi.1006695)
Supplement: S1 Table — Model name is shown at the top. The corresponding datasets can be identified by the stimulus frequencies tested: ORIG: 3, 4 and 6cpb. FREQ: 1, 3, 6, 12 and 24cpb. (PDF) [file pcbi.1006695.s001.pdf]

Table 1. Generative parameters fitted with the best four models.

Model KAm (ORIG)							
Adaptation type	Frequency	Learning rate K		Persistence rate A		Bias parameter m	
		mean	***SEM***	***mean***	***SEM***	***mean***	***SEM***
Two-way	3 cpb	0.0119	0.0027	0.9594	0.0110	- 0.0137	0.0041
	4 cpb	0.0109	0.0027	0.9599	0.0143	- 0.0156	0.0056
	6 cpb	0.0135	0.0039	0.9310	0.0266	- 0.0224	0.0080
Global	3 cpb	0.0052	0.0012	0.9733	0.0077	- 0.0145	0.0048
	4 cpb	0.0046	0.0007	0.9833	0.0051	- 0.0107	0.0040
	6 cpb	0.0045	0.0015	0.9786	0.0094	- 0.0117	0.0044
Model KAm (FREQ)							
Two-way	1 cpb	0.0121	0.0015	0.9734	0.0046	- 0.0128	0.0037
	3 cpb	0.0254	0.0087	0.9042	0.0342	- 0.0398	0.0204
	6 cpb	0.0217	0.0052	0.9014	0.0259	- 0.0382	0.0125
	12 cpb	0.0247	0.0079	0.8703	0.0629	- 0.0429	0.0260
	24 cpb	0.0374	0.0162	0.8337	0.1151	- 0.0450	0.0244
Global	1 cpb	0.0092	0.0024	0.9595	0.0201	- 0.0103	0.0030
	3 cpb	0.0103	0.0044	0.9595	0.0190	- 0.0224	0.0119
	6 cpb	0.0139	0.0068	0.8654	0.0703	- 0.0518	0.0272
	12 cpb	0.0153	0.0062	0.9123	0.0686	- 0.0325	0.0216
	24 cpb	0.0073	0.0038	0.9600	0.0184	- 0.0191	0.0077

Model $KAmG$ (ORIG)							
Adaptation type	Frequency	Learning rate K		Persistence rate A		Bias parameter m	
		mean	***SEM***	***mean***	***SEM***	***mean***	***SEM***
Two-way	3 cpb	0.0087	0.0023	0.9739	0.0109	- 0.0091	0.0039
	4 cpb	0.0078	0.0020	0.9785	0.0092	- 0.0118	0.0057
	6 cpb	0.0097	0.0028	0.9463	0.0258	- 0.0152	0.0070
Global	3 cpb	0.0045	0.0012	0.9832	0.0067	- 0.0087	0.0031
	4 cpb	0.0038	0.0008	0.9932	0.0026	- 0.0045	0.0015
	6 cpb	0.0049	0.0016	0.9794	0.0094	- 0.0101	0.0041
Model $KAmG$ (FREQ)							
Two-way	1 cpb	0.0103	0.0020	0.9811	0.0051	- 0.0081	0.0036
	3 cpb	0.0234	0.0097	0.8725	0.0842	- 0.0241	0.0090
	6 cpb	0.0132	0.0048	0.9638	0.0211	- 0.0147	0.0069
	12 cpb	0.0144	0.0052	0.9506	0.0365	- 0.0392	0.0262
	24 cpb	0.0334	0.0158	0.8293	0.1300	- 0.0645	0.0382
Global	1 cpb	0.0056	0.0006	0.9838	0.0032	- 0.0066	0.0021
	3 cpb	0.0056	0.0010	0.9819	0.0068	- 0.0096	0.0032
	6 cpb	0.0024	0.0023	1.0010	0.0121	- 0.0020	0.0065
	12 cpb	0.0139	0.0067	0.9195	0.0735	- 0.0286	0.0230
	24 cpb	0.0045	0.0036	0.9993	0.0044	- 0.0026	0.0010

Model $KmDG$ (ORIG)							
Adaptation type	Frequency	Learning rate K		Learning rate D		Bias parameter m	
		mean	***SEM***	***mean***	***SEM***	***mean***	***SEM***
Two-way	3 cpb	0.1575	0.0168	- 0.1541	0.0167	-0.0016	0.0003
	4 cpb	0.1351	0.0137	- 0.1313	0.0136	- 0.0017	0.0003
	6 cpb	0.1267	0.0173	- 0.1232	0.0170	- 0.0015	0.0003
Global	3 cpb	0.0667	0.0177	- 0.0644	0.0176	- 0.0013	0.0002
	4 cpb	0.0448	0.0109	- 0.0416	0.0107	- 0.0018	0.0005
	6 cpb	0.0349	0.0179	- 0.0320	0.0175	- 0.0012	0.0007
Model $KmDG$ (FREQ)							
Two-way	1 cpb	0.2235	0.0275	- 0.2210	0.0273	- 0.0013	0.0002
	3 cpb	0.1568	0.0308	- 0.1530	0.0308	- 0.0017	0.0004
	6 cpb	0.1233	0.0169	- 0.1171	0.0165	- 0.0026	0.0004
	12 cpb	0.0925	0.0101	- 0.0861	0.0107	- 0.0031	0.0007
	24 cpb	0.0831	0.0168	- 0.0751	0.0166	- 0.0035	0.0010
Global	1 cpb	0.1018	0.0167	- 0.0996	0.0168	- 0.0012	0.0002
	3 cpb	0.0733	0.0151	- 0.0697	0.0152	- 0.0019	0.0003
	6 cpb	0.0521	0.0134	- 0.0509	0.0132	- 0.0009	0.0007
	12 cpb	0.0310	0.0110	- 0.0251	0.0112	- 0.0019	0.0005
	24 cpb	0.0340	0.0095	- 0.0271	0.0086	- 0.0029	0.0009

Model $KAmDG$ (ORIG)									
Adaptation type	Frequency	Learning rate K		Learning rate D		Persistence rate A		Bias parameter m	
		Mean	***SEM***	***Mean***	***SEM***	***mean***	***SEM***	***mean***	***SEM***
Two-way	3 cpb	0.1552	0.0170	- 0.1519	0.0170	0.9994	0.0013	- 0.0020	0.0006
	4 cpb	0.1199	0.0206	- 0.1152	0.0215	0.9950	0.0063	- 0.0029	0.0014
	6 cpb	0.1255	0.0178	- 0.1216	0.0174	0.9973	0.0017	- 0.0022	0.0004
Global	3 cpb	0.0784	0.0197	- 0.0761	0.0196	1.0014	0.0033	- 0.0014	0.0015
	4 cpb	0.0254	0.0147	- 0.0215	0.0145	0.9892	0.0057	- 0.0081	0.0039
	6 cpb	0.0328	0.0166	- 0.0294	0.0161	0.9932	0.0032	- 0.0042	0.0011
Model $KAmDG$ (FREQ)									
Two-way	1 cpb	0.1642	0.0566	- 0.1588	0.0583	0.9914	0.0063	- 0.0058	0.0034
	3 cpb	0.1338	0.0263	- 0.1255	0.0257	0.9828	0.0086	- 0.0108	0.0061
	6 cpb	0.1177	0.0222	- 0.1099	0.0229	0.9902	0.0163	- 0.0086	0.0067
	12 cpb	0.0912	0.0105	- 0.0835	0.0103	0.9917	0.0077	- 0.0093	0.0052
	24 cpb	0.0850	0.0171	- 0.0735	0.0161	0.9976	0.0061	- 0.0069	0.0029
Global	1 cpb	0.0518	0.0567	- 0.0476	0.0581	0.9905	0.0085	- 0.0067	0.0035
	3 cpb	0.0505	0.0278	- 0.0452	0.0294	0.9883	0.0104	- 0.0095	0.0060
	6 cpb	0.0568	0.0159	- 0.0547	0.0154	0.9934	0.0086	- 0.0026	0.0033
	12 cpb	0.0319	0.0112	- 0.0254	0.0114	1.0017	0.0030	- 0.0015	0.0010
	24 cpb	0.0318	0.0094	- 0.0290	0.0085	0.9978	0.0045	- 0.0025	0.0010

Model name and dataset are shown at the top. The corresponding datasets can also be identified by the stimulus frequencies tested: ORIG: 3, 4 and 6cpb. FREQ: 1, 3, 6, 12 and 24cpb. The initial condition parameter is not reported.
