# Supplementary material for: A generative learning model for saccade adaptation
Source: PLoS Comput Biol. 2019 Aug 9;15(8):e1006695. doi: 10.1371/journal.pcbi.1006695 (PMC6703699; doi:10.1371/journal.pcbi.1006695)
Supplement: S2 Table — Repeated-measures ANOVA (2 X 3 on data from ORIG; 2 X 5 on data from FREQ) with factors type of adaptation and stimulus frequency was run on each of the four best models. Model name is shown at the side of the table and parameter names are on the top. The dataset is indicated in the cell at the upper left corner next the the parameter names. Highlights indicate the cases where the corresponding factor shows significant effects. (PDF) [file pcbi.1006695.s002.pdf]

Table 2. ANOVA results on the generative parameters fitted with the best four models.

Dataset FREQ		K		A		m		D		G		$\kappa = K + D$		$\eta = K - D$	
Model	Regressor	F	$p > F$	F	$p > F$	F	$p > F$	F	$p > F$	F	$p > F$	F	$p > F$	F	$p > F$
KAm	Freq	0.89	0.47	0.89	0.48	1.01	0.41	N/A		N/A		N/A		N/A	
	Type	8.07	0.01	1.96	0.19	1.22	0.29								
	Subj	1.84	0.22	2.85	0.13	2.96	0.10								
	Freq*Type	1.10	0.37	0.75	0.56	0.41	0.80								
	Freq*Subj	0.86	0.71	1.00	0.49	1.11	0.36								
	Type*Subj	1.05	0.42	0.57	0.85	0.54	0.88								
$KAmG$	Freq	1.18	0.33	0.68	0.61	1.35	0.27	N/A		0.10	0.98	N/A		N/A	
	Type	8.57	0.01	2.70	0.13	2.71	0.13			0.01	0.93				
	Subj	0.90	0.59	0.75	0.69	0.78	0.67			2.34	0.05				
	Freq*Type	1.30	0.28	1.16	0.34	1.18	0.33			1.68	0.17				
	Freq*Subj	0.80	0.78	1.04	0.44	1.05	0.43			1.75	0.03				
	Type*Subj	0.95	0.50	1.07	0.41	1.58	0.13			0.84	0.61				
$KmDG$	Freq	21.38	0.00	N/A		4.60	< 0.01	23.16	0.00	1.03	0.40	5.97	< 0.001	22.31	0.00
	Type	35.12	< 0.01			3.33	0.09	34.69	< 0.001	0.00	0.99	1.63	0.23	34.97	< 0.001
	Subj	2.81	0.05			2.45	0.10	2.84	0.05	13.62	< 0.001	0.90	0.59	2.83	0.05
	Freq*Type	2.16	0.09			1.13	0.35	2.29	0.07	0.83	0.51	0.92	0.46	2.23	0.09
	Freq*Subj	0.97	0.54			0.89	0.65	0.99	0.51	1.31	0.18	0.65	0.93	0.98	0.52
	Type*Subj	2.38	0.02			1.33	0.24	2.36	0.02	0.62	0.82	1.33	0.23	2.37	0.02
$KAmDG$	Freq	1.24	0.31	0.72	0.58	0.57	0.69	1.30	0.28	0.12	0.98	0.64	0.64	1.27	0.30
	Type	15.81	< 0.01	0.59	0.46	2.00	0.18	14.30	< 0.01	0.01	0.94	5.17	0.04	15.08	< 0.01
	Subj	1.81	0.25	1.81	0.27	1.68	0.22	1.96	0.24	3.65	< 0.01	0.62	0.80	1.89	0.25
	Freq*Type	0.34	0.85	0.13	0.97	0.39	0.81	0.38	0.82	2.03	0.10	1.85	0.14	0.36	0.84
	Freq*Subj	0.82	0.76	0.89	0.65	1.04	0.45	0.80	0.78	2.30	< 0.01	1.48	0.09	0.81	0.77
	Type*Subj	0.98	0.48	0.74	0.70	1.12	0.36	0.91	0.54	2.02	0.04	2.73	< 0.01	0.94	0.52

Dataset ORIG		K		A		m		D		G		$\kappa = K + D$		$\eta = K - D$	
Model	Regressor	F	$p > F$	F	$p > F$	F	$p > F$	F	$p > F$	F	$p > F$	F	$p > F$	F	$p > F$
KAm	Freq	0.23	0.80	0.74	0.49	0.36	0.70	N/A		N/A		N/A		N/A	
	Type	12.64	< 0.01	8.09	0.02	2.18	0.17								
	Subj	1.58	0.30	1.17	0.42	2.79	0.23								
	Freq*Type	0.26	0.77	1.09	0.36	0.70	0.51								
	Freq*Subj	0.90	0.59	1.43	0.23	0.97	0.53								
	Type*Subj	1.64	0.18	1.07	0.43	0.70	0.70								
$KAmG$	Freq	0.39	0.68	1.45	0.26	0.5	0.62	N/A		0.82	0.46	N/A		N/A	
	Type	12.66	< 0.01	6.61	0.03	5.77	0.04			4.97	0.05				
	Subj	2.13	0.10	1.49	0.25	2.35	0.16			3.10	0.14				
	Freq*Type	0.05	0.95	1.01	0.38	0.49	0.62			0.51	0.61				
	Freq*Subj	2.20	0.05	2.46	0.03	1.86	0.10			0.64	0.82				
	Type*Subj	1.58	0.20	1.06	0.43	0.37	0.93			1.56	0.20				
$KmDG$	Freq	5.31	0.02	N/A		0.62	0.55	5.24	0.02	0.37	0.70	0.64	0.54	5.27	0.02
	Type	36.94	< 0.001			0.35	0.57	37.57	< 0.001	6.07	0.04	2.45	0.15	37.26	< 0.001
	Subj	1.91	0.17			1.40	0.36	1.87	0.17	4.46	0.13	1.10	0.45	1.89	0.17
	Freq*Type	0.00	1.00			0.14	0.87	0.01	0.99	0.09	0.91	0.21	0.81	0.00	1.00
	Freq*Subj	1.25	0.32			1.10	0.42	1.35	0.27	0.64	0.82	1.54	0.18	1.30	0.29
	Type*Subj	4.31	< 0.01			1.09	0.41	4.29	< 0.01	1.20	0.35	1.24	0.33	4.30	< 0.01
$KAmDG$	Freq	3.82	0.04	1.62	0.23	2.26	0.13	3.82	0.04	0.07	0.94	1.88	0.18	3.82	0.04
	Type	30.75	< 0.001	1.10	0.32	2.09	0.18	31.03	< 0.001	3.65	0.09	2.19	0.17	30.90	< 0.001
	Subj	0.99	0.48	0.88	0.56	1.01	0.49	0.94	0.51	3.79	0.11	1.71	0.31	0.97	0.50
	Freq*Type	0.64	0.54	1.02	0.38	2.01	0.16	0.70	0.51	0.25	0.78	0.09	0.92	0.67	0.52
	Freq*Subj	4.07	< 0.01	2.61	0.02	1.60	0.16	4.47	< 0.01	0.69	0.78	1.14	0.39	4.27	< 0.01
	Type*Subj	5.15	< 0.01	1.09	0.42	1.69	0.16	5.19	< 0.01	1.42	0.25	1.74	0.67	5.18	< 0.01

Repeated-measures ANOVA (2 X 3 on data from ORIG; 2 X 5 on data from FREQ) with factors type of adaptation and stimulus frequency was run on each of the four best models. Model name is shown at the side of the table and parameter names are on the top. The dataset is indicated in the cell at the upper left corner next to the parameter names. Highlights indicate the cases where the corresponding factor reached significance level.
